# Natural Halloysite-Templated Synthesis of Highly Graphitic Boron-Doped Hollow Carbon Nanocapsule Webs

**DOI:** 10.3390/nano12142352

**Published:** 2022-07-09

**Authors:** Feng Chen, Lulu Ma, Bing Li, Peiwen Jiang, Zhimin Song, Lei Huang

**Affiliations:** 1 School of Environmental and Biological Engineering, Henan University of Engineering, Zhengzhou 451191, China; chenfeng871588@163.com (F.C.); malulu1001@163.com (L.M.); hngclb@126.com (B.L.); jiangpeiwen2016@163.com (P.J.); 2 School of Geosciences and Engineering, North China University of Water Resources and Electric Power, Zhengzhou 450046, China; 3 School of Environmental Science and Engineering, Guangzhou University, Guangzhou 510006, China

**Keywords:** halloysite, carbon nanocapsule, hollow structure, boron doping, DFT calculation

## Abstract

Hollow carbon nanocapsules have been attracting growing interest due to their fascinating characteristics and extensive potential applications. In this work, a novel natural halloysite-templated synthesis approach for highly graphitic boron-doped hollow carbon nanocapsule webs (B-HCNCWs) using glucose as the carbon source and boric acid as the heteroatom dopant was first reported. The formation process and physicochemical properties of B-HCNCWs were revealed by SEM, TEM, XRD, Raman, Brunauer–Emmett–Teller (BET), and XPS characterization techniques. The outcomes showed that the as-obtained B-HCNCWs with hollow nanocapsule network architecture had a specific surface area of 263 m^2^ g^−1^, a pore volume of 0.8 cm^3^ g^−1^, a high degree of graphitization (81.4%), graphite-like interplanar spacing (0.3370 nm), and B-containing functional groups (0.77 at%). The density function theory (DFT) calculation demonstrated that the adsorption energies of Li on B-HCNCWs were much higher than that of HCNCWs, which proved that B-doping in a carbon matrix could increase the lithium intercalation capacity.

## 1. Introduction

In recent years, the design and synthesis of hollow carbon nanocapsules have aroused more and more interest due to their fascinating characteristics such as large surface area, abundant porosity, high encapsulation ability, low density, enhanced permeability, good conductivity, and excellent thermal and chemical stability [1,2]. Generally speaking, the template method (hard and soft template) is identified as a conventional and high-efficiency approach to fabricating these hollow carbon nanostructures with well-controlled morphology. Previous studies have involved a great variety of templates, such as the SBA-15, AAO, metal-organic frameworks (MOF), F127, and so on [3,4]. However, these artificial templates are pre-fabricated, which is power-wasting, time-consuming, and uneconomic [4]. With a well-defined hollow nanotubular structure, natural halloysite (Hal), a kind of aluminosilicate clay mineral with the empirical formula of Al_2_Si_2_O_5_(OH)_4_·2H_2_O, has merits including cost-effectiveness, environment-friendliness, huge availability, and possession of abundant mesopores, and it is distributed extensively in China, Australia, the United States, New Zealand, and Brazil [3]. Hence, it should be a promising alternative template for the controlled synthesis of hollow carbon nanomaterials.

The incorporation of heteroatoms (such as N, S, B, and P) into the carbon skeletons can further improve the physicochemical properties of hollow carbon nanocapsules and thus tremendously broaden their application [1,5,6]. Among these heteroatoms, B element has been explored as a very promising candidate for enhancing the properties of carbonaceous materials, such as conductibility, adsorption ability, surface reactivity, and lithium intercalation capacity [7,8]. In addition, increasing the degree of graphitization of carbonaceous material has also attracted the attention of many scholars all over the world, owing to its many potential applications in the fields of potassium ion batteries and photocatalytic water splitting [9,10]. However, to the best of the current authors’ knowledge, a facile protocol to synthesize hollow carbon nanocapsules with a high degree of graphitization and B-doping using Hal as the template has not been reported in the literature.

Herein, we presented a natural Hal-templated synthesis technology for B-HCNCWs using glucose as the carbon precursor and boric acid as the doping agent via the hydrothermal reaction, followed by carbonization and graphitization treatment. The formation process and physicochemical properties of B-HCNCWs were then investigated in detail through a sequence of characterizations. With their hollow nanocapsule network architecture, high degree of graphitization, and B-containing functional groups, B-HCNCWs have enormous potential as electrode materials for energy storage, electrocatalytic applications, and conversion equipment. Moreover, the DFT calculation demonstrated the positive effect of B-doping in B-HCNCWs toward Li adsorption.

## 2. Materials and Methods

**Figure 1:**
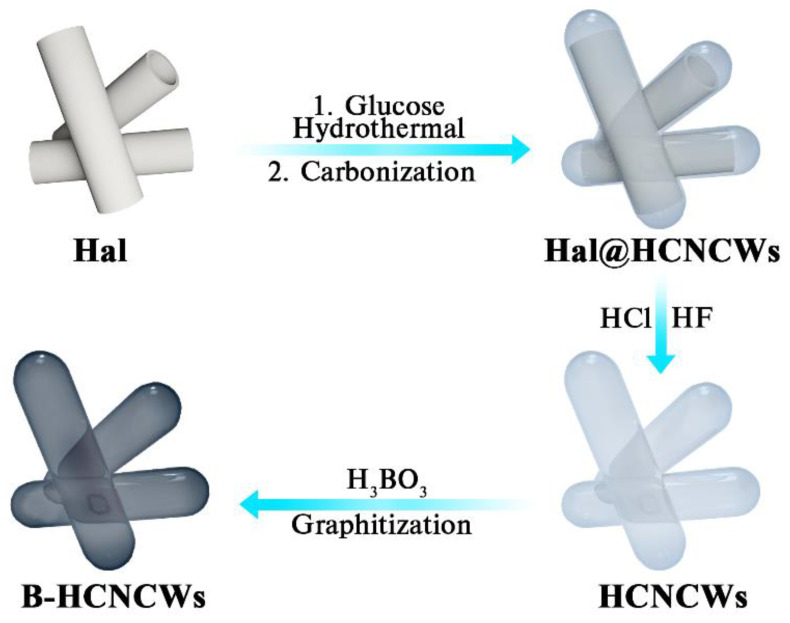
Schematic illustration for the synthesis of B-HCNCWs.

As displayed in Figure 1, the main synthetic process of B-HCNCWs comprised three steps: firstly, the HCNCWs-wrapped Hal (Hal@HCNCWs) composite was prepared by hydrothermal and carbonization treatment. Specifically, 0.6 g of Hal powder and 6 g of glucose were evenly dispersed into 70 mL of deionized water through ultrasonication for 1 h. The mixture was then moved into a 100 mL of PTFE-lined oxidation-resistant steel autoclave and maintained at 180 °C for 8 h. After cooling to indoor temperature, the as-prepared brown-black product was washed with deionized water and dehydrated ethanol a few times and then dried at 105 °C for 12 h in a vacuum drying oven, obtaining 2.1 g hydrothermal product. Afterward, the hydrothermal product (2.1 g) was put in a crucible and carbonized in a tubular furnace at 900 °C for 2 h under a nitrogen atmosphere with a heating speed of 5 °C min^−1^, and Hal@HCNCWs (1.2 g) was obtained after cooling. Secondly, the obtained Hal@HCNCWs was exposed to 1 M HCl and HF mixed solution and stirred continuously for 24 h to remove the Hal template. The samples were filtered and washed with deionized water until the pH = 7, and then dried at 60 °C for 24 h in a drying oven to obtain HCNCWs (0.53 g). Finally, 2 g HCNCWs and 0.4143 g H_3_BO_3_ (3 wt% B in the sample) were mixed evenly in an agate mortar for 30 min and placed in a graphite crucible, and thermally treated at 2600 °C for 30 min with a heating speed of 20 °C min^−1^ using a graphite furnace in an argon atmosphere. When naturally cooled to room temperature, the final B-HCNCWs (1.57 g) were obtained. The information on materials (the specifications and sources of reagents) and materials characterization (SEM, TEM, XRD, Raman, BET, and XPS) are described in detail in the Supplementary Materials. The density function theory (DFT) calculation was carried out using Materials Studio. The model for calculation was also built and applied in it using the Castep model with the quality of fine. The function was GGA/PBE. Monolayer graphene sheets containing different functional groups were extracted as the simplified models and then used as the initial structures of pristine carbon and boron-doped carbon materials. Therefore, the adsorption energy (E_ads_) of the most stable Li on the carbon materials was calculated by the following formula

(1) $$E_{ads}=E(C\cdots Li)-E(C)-E(Li)$$

where E(C⋯Li) was taken as the total energy of Li adsorbed on the surface of carbon, and E(C) and E(Li) referred to the energies of the free carbon and Li, respectively.

## 3. Results and Discussion

The micromorphologies and structures of natural Hal, Hal@HCNCWs, and B-HCNCWs were first characterized by scanning electron microscopy (SEM) and transmission electron microscopy (TEM) as displayed in Figures S1 and S2. From Figure S1a,b, it can be seen that natural Hal displayed the typical tubular nanostructures and the hollow lumens were readily surveyed. The length of Hal varies between a few hundred nanometers and a few micrometers, and the internal and external diameters ranged approximately from 5 to 20 and 20 to 50 nm, respectively. For Hal@HCNCWs, after the hydrothermal and high-temperature carbonization treatment, lots of carbon nanocapsules cross-linked with each other were observed as shown in Figure S1c. From Figure S1d, a thin amorphous carbon layer was found on the external surface of natural Hal, which was probably due to the adsorption of glucose molecules on the surface of Hal with the assistance of hydrogen bonding during the hydrothermal process followed by the transformation of the glucose layer into the carbon layer in the high-temperature carbonization procedure [11].

**Figure 2:**
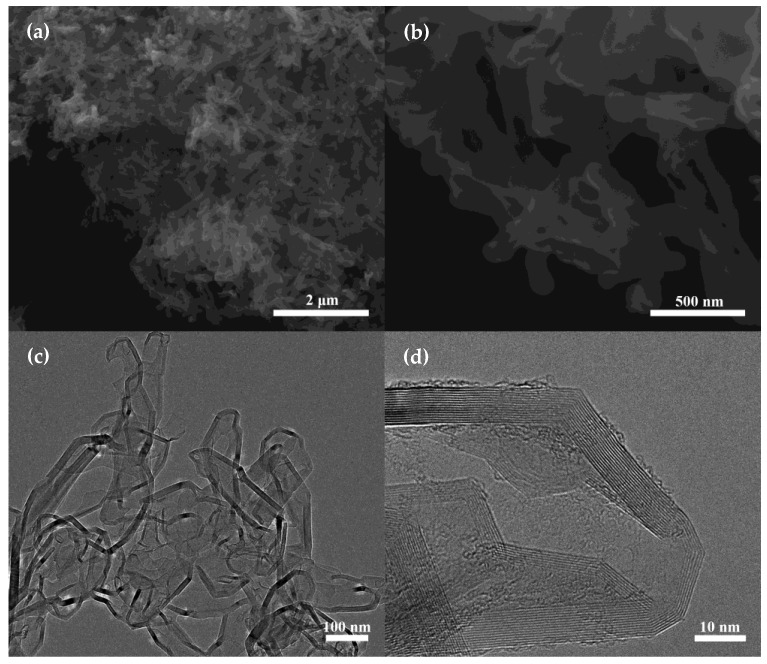
SEM (**a**,**b**) and TEM (**c**,**d**) images of B-HCNCWs.

For B-HCNCWs, as displayed in Figure 2a and Figure 2b, the tubular nanocapsule structure was surveyed and the nanocapsules interconnected with each other, forming particular porous three-dimensional webs. The length of each nanocapsule was similar to that of Hal. In addition, the TEM images of B-HCNCWs further confirmed their hollow nanocapsule structure (Figure 2c). The nanocapsules were kept very well with an outside diameter of approximately 80–120 nm after removing the Hal template. The high-resolution TEM (HRTEM) image of B-HCNCWs as shown in Figure 2d demonstrated that the carbon nanocapsules were made up of several to a dozen graphene layers, which is very similar to the microstructure of flake graphite, suggesting their high degree of graphitization [12]. The energy-dispersive spectrometer (EDS) elemental mapping of B-HCNCWs in Figure S2 verified the presence of boron (B), carbon (C), and oxygen (O) elements, which strongly proved that we had successfully synthesized boron-doped carbon materials.

**Figure 3:**
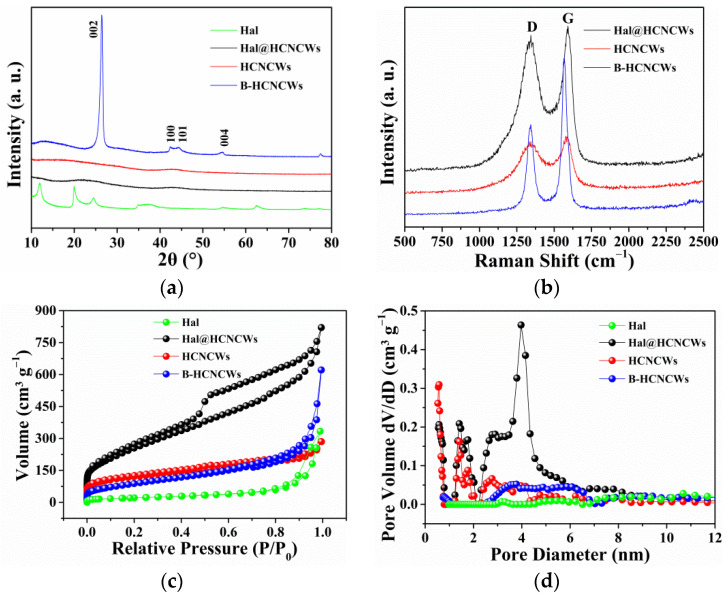
XRD patterns of Hal, Hal@HCNCWs, HCNCWs, and B-HCNCWs (**a**), Raman spectra (**b**) of Hal@HCNCWs, HCNCWs, and B-HCNCWs, nitrogen adsorption–desorption isotherms (**c**) and pore size distributions (**d**) of Hal, Hal@HCNCWs, HCNCWs, and B-HCNCWs.

Figure 3a and Figure S3 exhibited the XRD patterns of Hal, Hal@HCNCWs, HCNCWs, and B-HCNCWs. For Hal, the peaks located at 12.0°, 20.0°, 24.5°, 35.0°, 37.7°, 54.6°, and 62.4° were assigned to (001), (100), (002), (110), (003), (210), and (300) planes, respectively, which are the typical characteristic diffraction peaks of natural halloysite [13]. The diffraction peaks evident for Hal completely disappeared in the pattern of Hal@HCNCWs due to the deposition of the carbon coating layer on the surface of Hal. Two new broad peaks located in the range from 20° to 30° and from 40° to 50° were ascribed to the (002) and (100) lattice planes of graphite, respectively. The relatively broad and weak peak signals indicated the amorphous nature of the carbon coating layer, which consisted of small domains of ordered graphene sheets (Figure S3) [14]. After the removal of the Hal template, HCNCWs kept similar characteristic peaks to Hal@HCNCWs. For B-HCNCWs, four obvious and sharp characteristic peaks situated at 2θ = 26.4°, 42.4°, 44.5°, and 54.6° were observed, corresponding to the (002), (100), (101), and (004) planes of flake graphite [15], indicating that the amorphous carbon coating layer in HCNCWs turned into highly graphitic carbon in B-HCNCWs during the high-temperature graphitization process (2600 °C). In addition, the interplanar spacing (d_002_) of B-HCNCWs was calculated to be 0.3370 nm according to the Bragg equation, which was very close to that of flake graphite (0.3354 nm). The graphitization degree of B-HCNCWs also reached up to 81.4% based on the calculating formula reported in the literature [16].

The Raman spectra of Hal@HCNCWs, HCNCWs, and B-HCNCWs were shown in Figure 3b. The two distinct peaks at 1340–1350 cm^−1^ and 1565–1595 cm^−1^ in the spectra of the three samples represented the D band and G band, respectively. The D band was relevant to the defects, heteroatomic doping, and disorder induced in sp^3^-bonded carbon, while the G band corresponded to the in-plane vibration of sp^2^-hybridized carbon atoms of the crystalline graphite structure [3,15]. In addition, the relative strength ratio of the G band to D band (R = I_G_/I_D_) was generally used to describe the graphitization degree where a higher R value demonstrated a higher graphitic carbon content [14]. The R values of Hal@HCNCWs, HCNCWs, and B-HCNCWs were 1.06, 1.09, and 1.72, respectively. The super high R value of B-HCNCWs indicated its high graphitization degree, which was consistent with the HRTEM and XRD results.

The nitrogen adsorption–desorption isotherms of Hal, Hal@HCNCWs, HCNCWs, and B-HCNCWs were displayed in Figure 3c. The isotherms of the four specimens all belonged to the integration of type I/IV isotherms with hysteresis cycles, suggesting their microporous and mesoporous characteristics [12]. The sharp increase in N_2_ adsorption at a low relative pressure of P/P_0_ < 0.1 was normally related to the filling of micropores. The N_2_ sorption volume (P/P_0_ < 0.1) of Hal was very small, indicating that the micropores were almost negligible. Furthermore, the N_2_ sorption volume (P/P_0_ < 0.1) of Hal@HCNCWs was larger than those of HCNCWs and B-HCNCWs, suggesting the larger microporous volume of Hal@HCNCWs. The hysteresis loop at a relative pressure (P/P_0_) in the scope of 0.5–1 for Hal@HCNCWs was also the most obvious, indicating its largest mesoporous volume among the four samples.

The resulting pore size distribution patterns of Hal, Hal@HCNCWs, HCNCWs, and B-HCNCWs calculated from the N_2_ adsorption data according to the DFT method were then shown in Figure 3d and Figure S4. For Hal, we can see that the pore sizes possessed the distribution between 2.5 and 25 nm and were centered at 3.2, 5.4, 8.4, 10.7, and 14.7 nm, indicating that Hal mainly contained mesoporous (Figure S4). For Hal@HCNCWs, two areas of micropores with sizes of 0.5–0.8 nm and 1.2–2.0 nm and a distinct peak centered at 4.0 nm could be surveyed, indicating that Hal@HCNCWs contained both micro- and mesopores. The HCNCWs displayed a similar area of micropores to Hal@HCNCWs except that the area of mesopores with the size of 2.0–5.6 nm disappeared, which was attributed to the etching of the Hal template. For B-HCNCWs, we can see that it contained almost negligible micropores below 1.0 nm and some mesopores between 2.4 and 7.0 nm, and few mesopores between 7.0 and 25 nm; this was because the high-temperature graphitization reduced the number of pores. Table S1 summarized the textural parameters of Hal, Hal@HCNCWs, HCNCWs, and B-HCNCWs. The corresponding specific surface areas of Hal, Hal@HCNCWs, HCNCWs, and B-HCNCWs were, respectively, 77, 920, 400, and 263 m^2^ g^−1^. The pore volumes of Hal, Hal@HCNCWs, HCNCWs, and B-HCNCWs were, respectively, 0.5, 1.0, 0.3, and 0.8 cm^3^ g^−1^. It is worth noting that the portion of specific surface area and porosity for B-HCNCWs was still retained even after heating at 2600 °C; such a unique textural property was conducive to the extension of its application.

**Figure 4:**
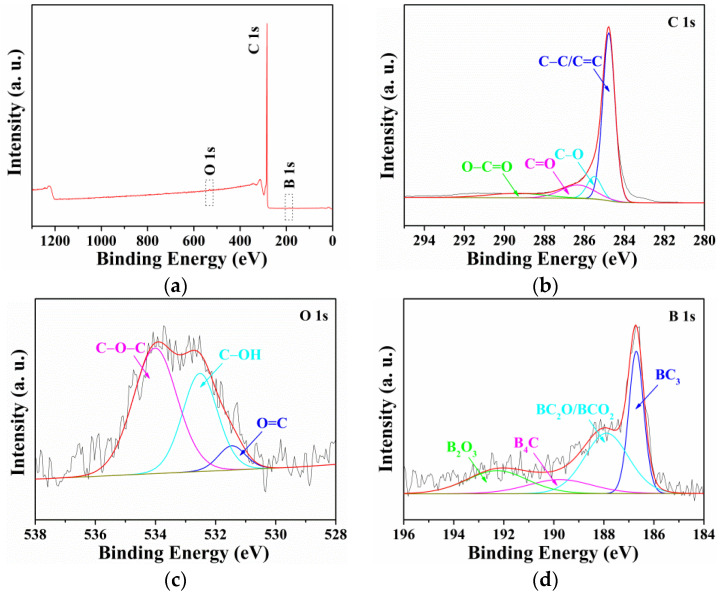
The XPS survey spectrum of B-HCNCWs (**a**), the high-resolution C 1s (**b**), O 1s (**c**), and B 1s (**d**) XPS spectra of B-HCNCWs.

Further evidence for the surface chemical compositions and states of Hal@HCNCWs, HCNCWs, and B-HCNCWs was obtained by XPS spectra as displayed in Figure 4, Figure S5 and S6. As exhibited in Figure S5a, the Hal@HCNCWs contained C, O, Si, and Al elements, and the peaks for Si and Al were not apparent due to the HCNCWs carbon coating on the outside surface of Hal. After the removal of Hal, the XPS spectrum of HCNCWs only contained C 1s and O 1s (Figure S6a). The XPS spectrum of B-HCNCWs revealed the peaks for C 1s, O 1s, and B 1s (Figure 4a), and elemental contents obtained from XPS analysis for Hal@HCNCWs, HCNCWs, and B-HCNCWs were summarized in Table S2. We can see that the contents of C, O, and B elements for B-HCNCWs were 98.58, 0.65, and 0.77 at%, respectively, indicating that B-doped carbon materials had been successfully prepared, which was in good agreement with the outcomes of EDS elemental mapping. Compared with Hal@HCNCWs and HCNCWs, the C content (98.58 at%) increased while the O content (0.65 at%) decreased for B-HCNCWs, again demonstrating its highly graphitized structure.

As shown in Figure 4b, four peaks of B-HCNCWs centered at 284.8, 285.5, 286.3, and 289.4 eV were subdivided from the high-resolution C 1s spectrum, corresponding to C–C/C=C, C–O, C=O, and O–C=O, respectively [17]. The content of non-oxygenated C–C/C=C groups was as high as 66.43%, suggesting a high graphitization degree of B-HCNCWs, which was in accordance with the outcomes of the XRD and Raman analyses. The high-resolution O 1s spectrum could be fitted to three peaks located at 531.5, 532.5, and 534.0 eV, belonging to O=C, C–OH, and C–O–C, respectively (Figure 4c) [17]. The residual oxygen-containing functional groups could introduce some radicals on the surfaces of B-HCNCWs, which would greatly improve their physicochemical properties. According to the high-resolution B 1s XPS spectrum (Figure 4d), B was present in B-HCNCWs mainly as four types of B-species: BC_3_ (186.7 eV), BC_2_O/BCO_2_ (187.9 eV), B_4_C (189.8 eV), and B_2_O_3_ (192.3 eV) [4,18,19]. The XPS analysis suggested that B-doping has modified the surface chemistry of B-HCNCWs, which showed a good prospect in the application of the electrode materials.

**Figure 5:**
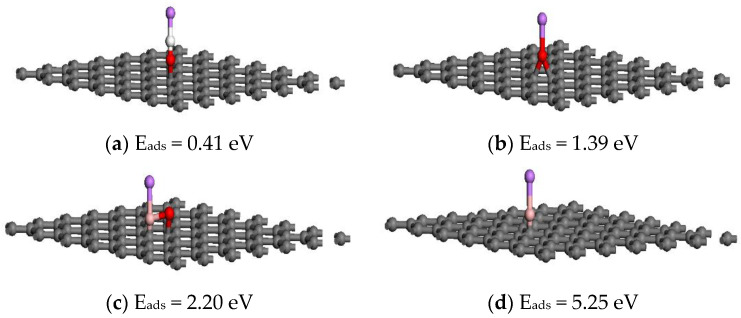
The optimized geometries and corresponding adsorption energies of the most stable Li on HCNCWs with OH (**a**), O (**b**), BO (**c**), and B (**d**) species. The gray, white, red, pink and purple balls represent C, H, O, B, and Li atoms, respectively.

In order to further verify the potential application of B-HCNCWs as electrode materials, the DFT calculation was carried out. The optimized geometries and corresponding adsorption energies of the most stable Li on HCNCWs with OH, O, BO, and B species were shown in Figure 5. We can see that the adsorption energies of Li on HCNCWs with OH, O, BO, and B species were 0.41, 1.39, 2.20, and 5.25 eV, respectively. Notably, the adsorption energies of Li on HCNCWs with BO and B species (that was B-HCNCWs) were all much higher than those of HCNCWs with OH and O species, indicating the positive effect of B-doping in the carbon matrix toward Li adsorption. Therefore, it was reasonable to conclude that B-doping in the carbon matrix could increase the lithium intercalation capacity, which also proved that B-HCNCWs have a bright prospect as the electrode materials in lithium-ion batteries. The results of the DFT calculation also verified the experimental conclusions reported in the literature [20].

## 4. Conclusions

In summary, B-HCNCWs were successfully synthesized via the hydrothermal reaction followed by carbonization and graphitization treatment using natural Hal as the template. The forming process and physicochemical properties of B-HCNCWs were confirmed by a sequence of characterizations. Since B-HCNCWs have hollow nanocapsule network architecture, a high degree of graphitization, graphite-like interplanar spacing, and B-containing functional groups, they could be extensively applied in the fields of electrode materials, adsorbents, catalysts, and sensors. Moreover, the DFT calculation demonstrated that B-doping in the B-HCNCWs matrix could increase the lithium intercalation capacity.

## Data Availability

Not applicable.

## Supplementary Materials

The following are available online at https://www.mdpi.com/article/10.3390/nano12142352/s1, Figure S1: SEM (a) and TEM (b) images of natural Hal, SEM (c) and TEM (d) images of Hal@HCNCWs, Figure S2: EDS elemental maps of boron (yellow), carbon (purple), and oxygen (green) for B-HCNCWs, Figure S3: XRD patterns of Hal, Hal@HCNCWs and HCNCWs, Figure S4: pore size distributions of Hal and B-HCNCWs, Figure S5: The XPS survey spectrum of Hal@HCNCWs (a), the high-resolution C 1s (b), O 1s (c), Al 2p (d) and Si 2p (e) XPS spectra of Hal@HCNCWs, Figure S6: The XPS survey spectrum of HCNCWs (a), the high-resolution C 1s (b) and O 1s (c) XPS spectra of HCNCWs, Table S1: The textural parameters of Hal, Hal@HCNCWs, HCNCWs, and B-HCNCWs, Table S2: Elemental contents obtained from XPS analysis for Hal@HCNCWs, HCNCWs, and B-HCNCWs.
