# Supplementary material for: Natural Halloysite-Templated Synthesis of Highly Graphitic Boron-Doped Hollow Carbon Nanocapsule Webs
Source: Nanomaterials (Basel). 2022 Jul 9;12(14):2352. doi: 10.3390/nano12142352 (PMC9316536; doi:10.3390/nano12142352)
Supplement: Supplementary file 1 [file nanomaterials-12-02352-s001.zip › nanomaterials-1800815-supplementary.pdf]

Supplementary Materials

Natural Halloysite-Templated Synthesis of Highly Graphitic Boron-Doped Hollow Carbon Nanocapsule Webs

Feng Chen [1], Lulu Ma [1], Bing Li [1], Peiwen Jiang [1], Zhimin Song [1,2,*] and Lei Huang [3,*]

[1] School of Environmental and Biological Engineering, Henan University of Engineering, No. 1, Xianghe Road, Zhengzhou 451191, China; chenfeng871588@163.com (F.C.); malulu1001@163.com (L.M.); hngclb@126.com (B.L.); jiangpeiwen2016@163.com (P.J.)

[2] School of Geosciences and Engineering, North China University of Water Resources and Electric Power, Zhengzhou 450046, China

[3] School of Environmental Science and Engineering, Guangzhou University, Guangzhou 510006, China

* Correspondence: songzhimin1961@hotmail.com (Z.S.); huanglei@gzhu.edu.cn (L.H.); Tel.: +86-037162508218 (Z.S.); +86-073188830875 (L.H.)

1. Supplementary Experimental

1.1 Materials

Natural halloysite clay was directly excavated in Changsha, Hunan province in China. Before the experiment, the obtained halloysite clay was first washed with deionized water to eliminate the deposition of nonsoluble clay. Halloysite was then collected through filtration, washed with deionized water, and dried at 60 °C for 24 h in a vacuum drying oven. Glucose ($C_6H_{12}O_6$), boric acid (H_3BO_3), and dehydrated ethanol (C_2H_5OH, 99.5%) were provided by Xilong Chemical Co., Ltd (Wuhan, China). Hydrochloric acid (HCl, 36.0–38.0 wt%) and hydrofluoric acid (HF, 40.0 wt%) were purchased from Luoyang Haohua Chemical Reagent Co., Ltd (Luoyang, China). The water used in the experiment was high purity deionized water, and all the reagents were used as received without any further purification.

1.2 Materials Characterization

The structures and micromorphologies of the obtained samples were characterized by scanning electron microscopy (SEM, Quanta FEG 250, Tokyo, Japan) with an energy dispersive spectrometer (EDS) and field-emission transmission electron microscopy (TEM, JEM-2100F, Tokyo, Japan). The crystal patterns were observed by powder X-ray diffraction (XRD, D8 Advance, Karlsruhe, Germany) using Cu K_{α} (λ=1.5418 Å) radiation within the scope of 10°–80°. The Raman spectroscopy was recorded by a Raman scattering instrument (in Via Reflex, Renishaw, London, UK) with a wavelength of 514 nm and a wave number range of 500–2500 cm^{-1}. The N_2 adsorption–desorption isotherms were obtained by a specific surface area and porosity analyzer (Autosorb iQ 2 MP-XR, Boynton Beach, USA) at the liquid nitrogen condition. The Brunauer–Emmett–Teller (BET) method and Barrett–Joyner–Halenda (BJH) method were used to calculate the specific surface area and pore size distribution of the samples according to the nitrogen adsorption data, respectively. The X-ray photoelectron spectroscopy (XPS, Thermo ESCALAB 250XI, Waltham, USA) tests were implemented to analyze the surface chemical composition and states of the samples.

2. Supplementary Figures

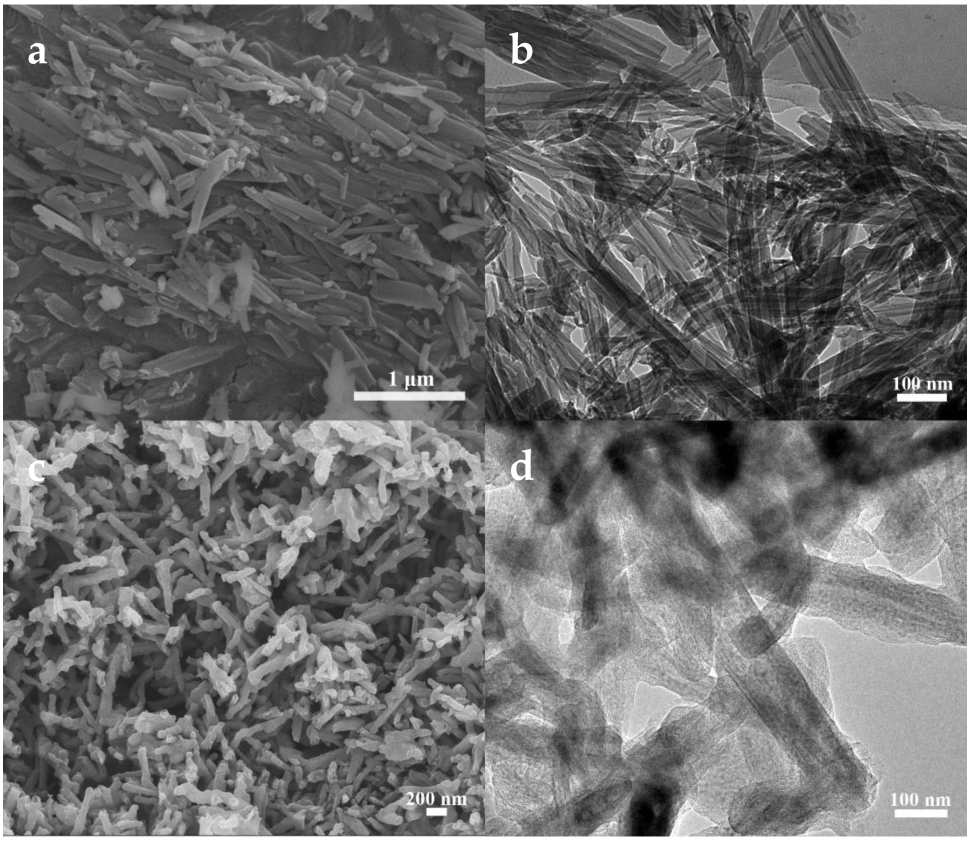

Figure S1. SEM (a) and TEM (b) images of natural Hal, SEM (c) and TEM (d) images of Hal@HCNCWs.

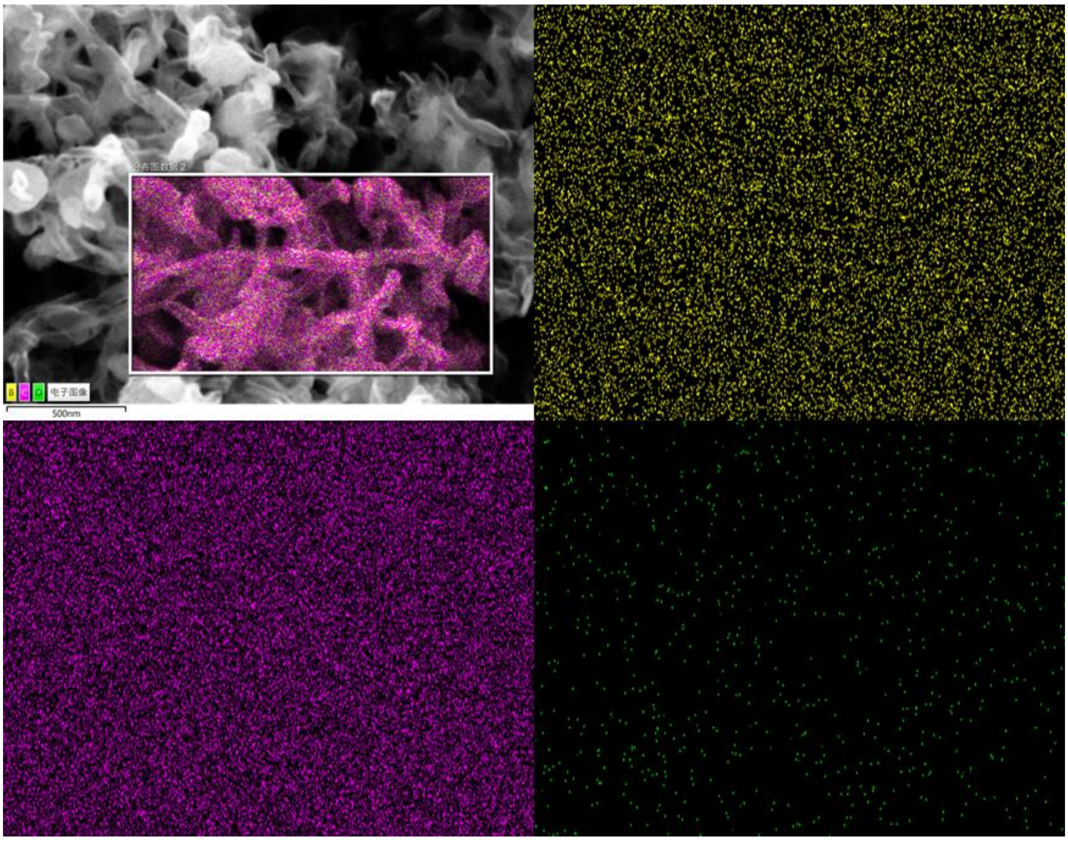

Figure S2. EDS elemental maps of boron (yellow), carbon (purple), and oxygen (green) for B-HCNCWs.

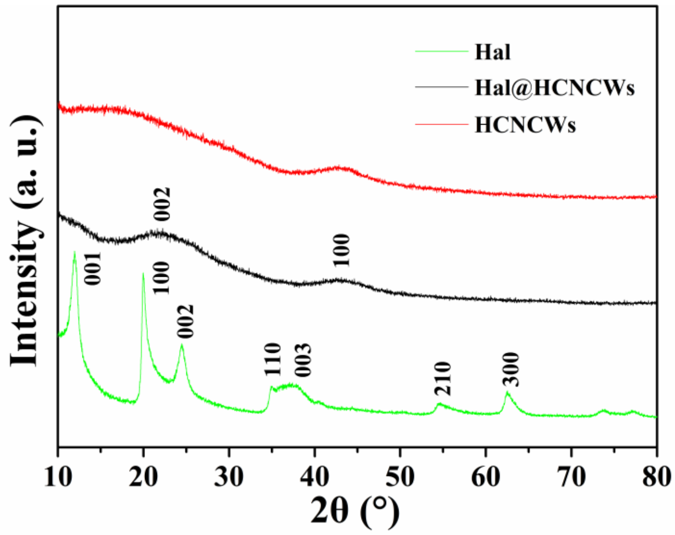

Figure S3. XRD patterns of Hal, Hal@HCNCWs, and HCNCWs.

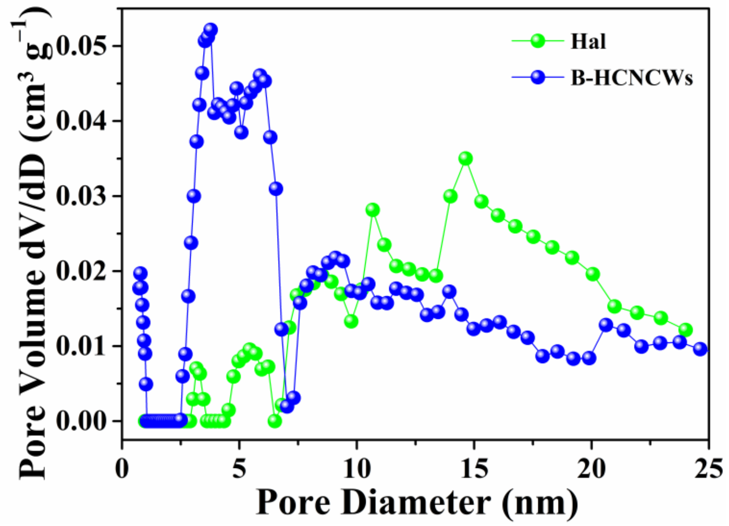

Figure S4. Pore size distributions of Hal and B-HCNCWs.

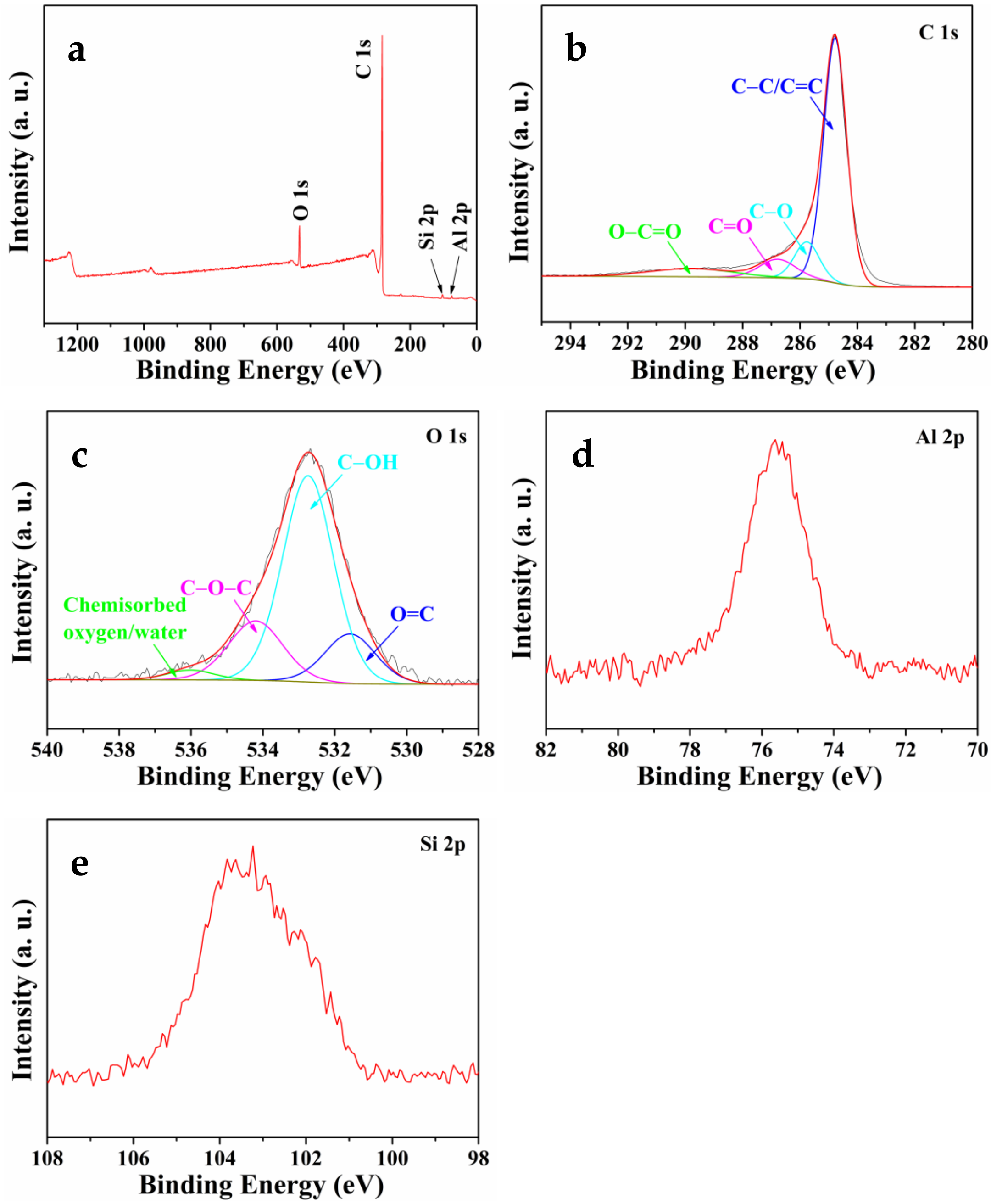

Figure S5. The XPS survey spectrum of Hal@HCNCWs (a), the high-resolution C 1s (b), O 1s (c), Al 2p (d), and Si 2p (e) XPS spectra of Hal@HCNCWs.

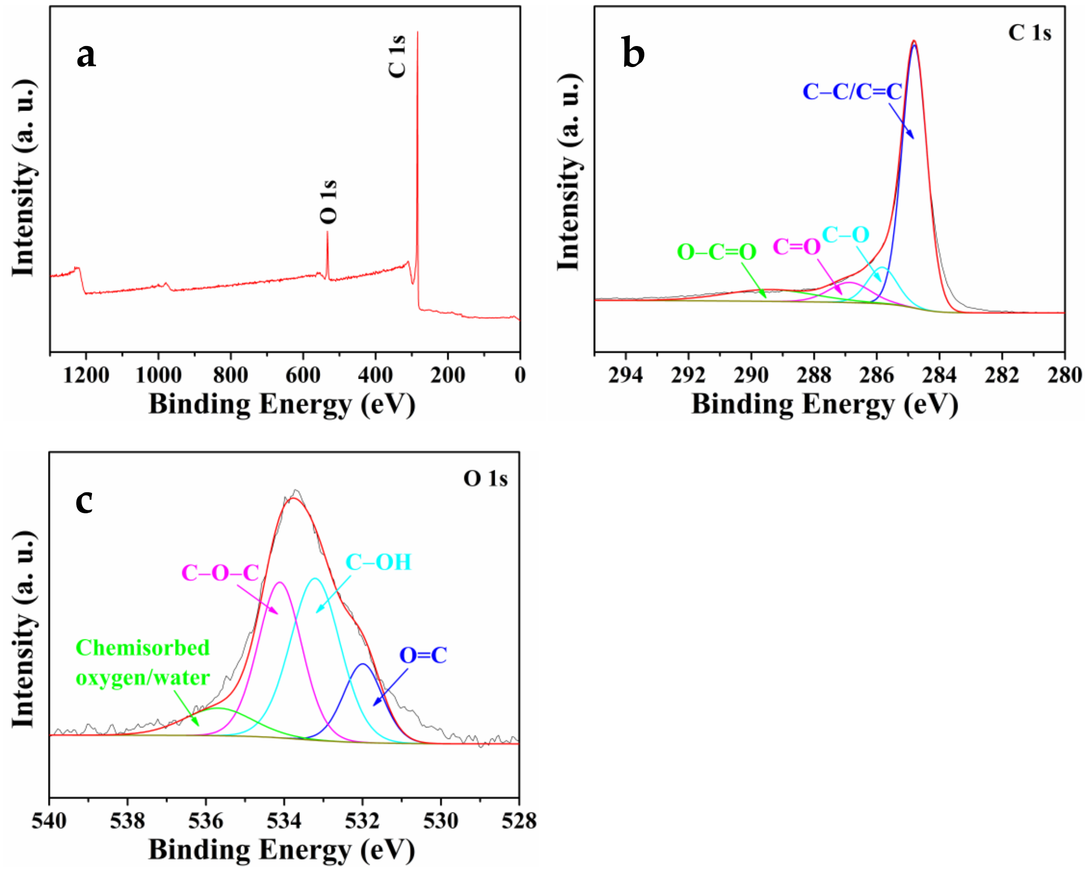

Figure S6. The XPS survey spectrum of HCNCWs (a), the high-resolution C 1s (b), and O 1s (c) XPS spectra of HCNCWs.

3. Supplementary Table

Table S1 The textural parameters of Hal, Hal@HCNCWs, HCNCWs, and B-HCNCWs.

Parameters	Samples			
	Hal	Hal@HCNCWs	HCNCWs	B-HCNCWs
Specific surface area ($m^2\ g^{-1}$)	77	920	400	263
Pore volume ($cm^3\ g^{-1}$)	0.5	1.0	0.3	0.8
Average pore diameter (nm)	15.7	4.0	0.6	3.8

Table S2 Elemental contents obtained from XPS analysis for Hal@HCNCWs, HCNCWs, and B-HCNCWs.

Samples	C (at%)	O (at%)	B (at%)	Al (at%)	Si (at%)
Hal@HCNCWs	89.2	7.67	0	1.39	1.74
HCNCWs	91.14	8.86	0	0	0
B-HCNCWs	98.58	0.65	0.77	0	0
